# The Role of Polyphenols in Regulation of Heat Shock Proteins and Gut Microbiota in Weaning Stress

**DOI:** 10.1155/2021/6676444

**Published:** 2021-09-06

**Authors:** Tarique Hussain, Jing Wang, Ghulam Murtaza, Elsayed Metwally, Huansheng Yang, Muhammad Saleem Kalhoro, Dildar Hussain Kalhoro, Baban Ali Rahu, Bie Tan, Raja Ghazanfar Ali Sahito, Muhammad Ismail Chughtai, Yulong Yin

**Affiliations:** ^1^College of Animal Science and Technology, Hunan Agricultural University, Changsha, 410128 Hunan, China; ^2^Animal Science Division, Nuclear Institute for Agriculture and Biology College, Pakistan Institute of Engineering and Applied Sciences (NIAB-C, PIEAS), Faisalabad 38000, Pakistan; ^3^Department of Animal Reproduction, Faculty of Animal Husbandry and Veterinary Sciences, Sindh Agriculture University, Tandojam, Sindh 70050, Pakistan; ^4^Department of Cytology & Histology, Faculty of Veterinary Medicine, Suez Canal University, Ismailia, Egypt; ^5^Hunan International Joint Laboratory of Animal Intestinal Ecology and Health, Laboratory of Animal Nutrition and Human Health, College of Life Sciences, Hunan Normal University, Changsha, Hunan 410081, China; ^6^Department of Animal Products Technology, Faculty of Animal Husbandry and Veterinary Sciences, Sindh Agriculture University, Tandojam, Sindh 70050, Pakistan; ^7^Department of Veterinary Microbiology, Faculty of Animal Husbandry and Veterinary Sciences, Sindh Agriculture University, Tandojam, Sindh 70050, Pakistan; ^8^Institute of Neurophysiology, University of Cologne, Cologne 50931, Germany; ^9^Institute of Subtropical Agriculture, Chinese Academy of Sciences, Changsha, 410125 Hunan, China

## Abstract

Gut microbiota is the natural residents of the intestinal ecosystem which display multiple functions that provide beneficial effects on host physiology. Disturbances in gut microbiota in weaning stress are regulated by the immune system and oxidative stress-related protein pathways. Weaning stress also alters gut microbiota response, limits digestibility, and influences animal productive performance through the production of inflammatory molecules. Heat shock proteins are the molecular chaperones that perform array functions from physiological to pathological point of view and remodeling cellular stress response. As it is involved in the defense mechanism, polyphenols ensure cellular tolerance against enormous stimuli. Polyphenols are nature-blessed compounds that show their existence in plenty of amounts. Due to their wider availability and popularity, they can exert strong immunomodulatory, antioxidative, and anti-inflammatory activities. Their promising health-promoting effects have been demonstrated in different cellular and animal studies. Dietary interventions with polyphenols may alter the gut microbiome response and attenuate the weaning stress related to inflammation. Further, polyphenols elicit health-favored effects through ameliorating inflammatory processes to improve digestibility and thereby exert a protective effect on animal production. Here, in this article, we will expand the role of dietary polyphenol intervention strategies in weaning stress which perturbs gut microbiota function and also paid emphasis to heat shock proteins in gut health. This review article gives new direction to the feed industry to formulate diet containing polyphenols which would have a significant impact on animal health.

## 1. Introduction

Gut microbiota is the natural inhabitant of the gastrointestinal tract, residing in a host with mutual understanding over the decades [1]. It performs a well-maintained composition and balance within the host homeostasis [2]. The gut microbiota of pigs represents vibrant composition and diversity that alters with time and throughout the gastrointestinal tract [3]. The initiation of microbial colonization starts to develop at birth and is shaped by the consumption of sow's milk [4], and thus, the suckling period indicates gut microbial alterations. The weaning period especially starts from 3 to 4 weeks after sowing, when piglets have been offered a solid diet instead of liquid milk. Weaned piglets are sensitive to nutritional, physiological, and psychological stressors, leading to disrupted intestinal morphology, physiological function, and shifts in gut microbiota [5, 6]. The alteration in gut microbiota is regarded as the main factor for postweaning diarrhea [7]. The prime function of gut microbiota is to digest indigestible nutrients, but it also helps in nutrient absorption, metabolism, and storage of ingested nutrients which has an influential impact on host physiology [8]. Disruption in the shift of gut microbiota composition may lead to several pathogenic disorders [9, 10]. Therefore, enhancement in host health needs a clear understanding of the intestinal ecosystem particularly gut microbiota [11].

It has been well understood that overwhelming free radicals induce oxidative stress, compromise scavenging free radicals, and influence oxidant/antioxidant balance. Oxidative stress also communicates with signaling molecules to exert a physiopathogenic response. Overall, it has a pronounced effect on gut health in pigs and beef calves which ultimately minimizes its production [12, 13]. Research on gut microbiota in pigs would not only help construct a healthy gut of the animal but also benefit human studies due to the similarity of anatomic and physiologic attributes. The implication of polyphenols shapes the gut microbiota, ameliorating weaning stress which is discussed in the following sections.

## 2. The Metabolism of Polyphenols by Gut Microbiota

**Figure 1:**
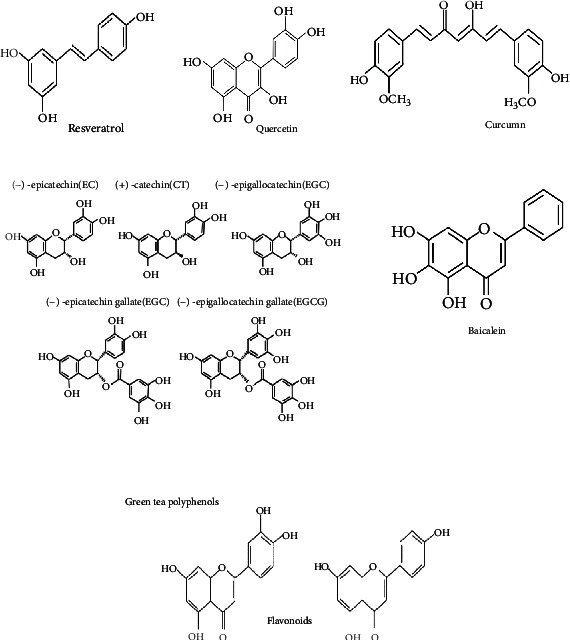
Chemical structures of polyphenols exerting health beneficial effects.

Dietary polyphenols are nature-based bioactive compounds derived from a variety of sources comprising plants, fruit, vegetables, cocoa, chocolate, tea, coffee, and wine [14]. Naturally, they are heterogeneous compounds categorized into hydroxylated phenyl moieties. Due to their different structural features, they have been distributed into flavonoids and nonflavonoids [15]. The majority of the compounds fall under the category of flavonoids; more than 9000 structurally different compounds have been investigated in nature so far. The unique identity of the phenolic compounds consists of a diphenylpropane skeleton (C6-C3-C6) structure with common attributes. Moreover, flavonoids are further subclassified into six subgroups according to their diverse structural behavior [16, 17]. Once these compounds have been consumed, the human body recognizes them as xenobiotics; therefore, the bioavailability of the compounds is lower than micro- and macronutrients. Considering the nature of the compound, they quickly pass through the small intestine [18] or move towards the colon nearly unchanged [19, 20]. The data has revealed that about 1/9 of total polyphenol absorption takes place in the small intestine, while the rest is transferred through the large intestinal lumen at a lower concentration, where they are conjugated and excreted via the intestinal lumen through bile and exposed to enzymatic activities [21–23]. Larger molecules of polyphenols reach the colon in an almost original structure and then metabolize through the action of gut microbiota along with conjugates and are eliminated in the intestinal lumen via bile. There are several factors which influence their bioavailability, such as polarity, molecular mass, plant matrix, digestibility, and absorption [24]. Once the polyphenols are absorbed from the small intestine, aglycones pass the biotransformation in enterocytes and then hepatocytes. After that, produced metabolites are disseminated to different organs and finally eliminated by urine. And scientists indicated that evaluations of the effects of polyphenol-rich foods on human blood antioxidant capacity might not consider the volunteers' food intake, which may serve as the major influence in affecting the blood antioxidant capacity of humans [25]. Further, comprehensive knowledge on the bioavailability of polyphenols is discussed by [26–30]. The polyphenol structure showing health protective effects is well illustrated in Figure 1.

## 3. The Effects of Polyphenols on Microbial Composition and Metabolism

The gastrointestinal tract (GIT) is inhabited by numerous species of bacteria in the colon. The predominantly found microbiota phyla are *Firmicutes*, *Bacteroidetes*, *Proteobacteria*, etc. [31]. The specific microbiota composition may vary in some conditions, such as diarrhea and antibiotic therapy, or with the nutritional intervention [32]. Diet impacts the gut microbiota and may alter the significance of well-being either with favorable or detrimental concerns. *Prevotella*, as the main bacteria in the gut system, is responsible for diet enriched in carbohydrates while Bacteroides is responsible for uptake of diet rich in animal protein and saturated fat [33, 34]. Few bacteria specifically *Flavonifractor plautii*, *Slackia equolifaciens*, and *Slackia isoflavoniconvertens* take part in the metabolism of many polyphenols. The dietary polyphenol intake is roughly calculated as more than 1 g that is 10 times greater than vitamin C intake [35]. The association between polyphenols and gut microbiome (GM) has been described somewhere else by [36].

The polyphenol mechanism regarding modification of gut microbiota needs to be rectified and serve its function through a direct or indirect way. They either involve in activation or suppression of bacterial growth. Suppressed bacterial growth defines the bacteriostatic or bactericidal effect of polyphenols that prevents the development of active pathogenic bacteria. Therefore, it is pivotal to deliberate the level and characteristics of these compounds. The indirect effect of polyphenol metabolites may trigger the development of one group of bacteria via promoting the growth of another group of bacteria [37, 38]. The regulation of polyphenol intake on the abundance and diversity of gut microbiota may be associated with the variety of substrate preferences and metabolic abilities of the gut microbial community [39]. Polyphenol can affect the *Firmicutes*/*Bacteroidetes* (F/B) ratio via suppression of particular bacterial species [40]. A randomized, double-blind, placebo-controlled human trial indicated that oral consumption of epigallocatechin-3-gallate and resveratrol at 282 and 80 mg/day, respectively, for 12 weeks positively reduced fecal abundance of *Bacteroidetes* and *Faecalibacterium prausnitzii* in obese individuals in response to placebo [41]. Rats exposed to dietary intake of quercetin at 30 mg/kg/day inhibited gut microbiota impairment triggered by high-fat diet via reducing the F/B ratio and declining the abundance of obese-related bacteria, for instance, *Erysipelotrichaceae*, *Bacillus*, and *Eubacterium cylindroides* [42]. Induction of polyphenol-rich foods/extracts also modified the composition of gut microbiota. The canine offered green tea polyphenol extracts for 18 weeks suppressed abundances of Bacteroidetes and Fusobacteria and enhanced the Firmicutes [43]. The mice treated with dietary anthocyanins at 5% and 10% freeze-dried black raspberry powder and challenged with azoxymethane/DSS for 12 weeks promoted fecal abundance of beneficial bacteria, for example, *Faecalibacterium prausnitzii*, *Lactobacillus*, and *Eubacterium rectale*, and declined the abundance of pathogens, such as *Desulfovibrio* sp. and *Enterococcus* spp. [44]. The most recent finding showed that exposure of wild blueberry polyphenolic extract and blueberries isolated fraction to high fat-sucrose diet augmented the growth of polyphenol degrading bacteria *Adlercreutzia equolifaciens* in obese mice, indicating that addition of these bacteria in polyphenol metabolism may be involved in mitigation of metabolic disorders in obesity and diabetes via using bioactive molecules [45]. Generally, the structure of polyphenols, optimizing doses, and strain of microorganisms may impact polyphenol effect on bacterial growth and metabolism. Polyphenols may increase the enriches of helpful bacteria, for instance, *Bifidobacterium* and *Lactobacillus* that protect gut barrier function, *Faecalibacterium prausnitzii* that indicates anti-inflammation effect via suppressing nuclear factor-kappa B (NF-*κ*B) stimulation, and *Roseburia* spp. that are butyric acid producers [46]. This study showed that Gram-positive bacteria are vulnerable to polyphenols against Gram-negative bacteria. These changes could be due to the difference in the cell wall composition of the bacteria [47].

## 4. Heat Shock Proteins and Gut Health

Heat shock proteins (HSPs) are a huge family of molecular chaperones, which could confirm the folding, unfolding, and refolding of stress-denatured proteins [48]. The HSPs are classified into seven families depending upon their molecular weight [49]. Most of the members act as a chaperone, stabilizing to correct protein or supporting in refolding proteins which are damaged by stress cell response [50]. HSPs serve essential functions in immune responses and tend to maintain mucosal barrier integrity and gut homeostasis. It becomes witnessed that enteric microbiota is considered the main inducers of heat shock protein production in intestinal epithelial cells [51]. They regulate gut barrier function through regulating tight junction proteins (TJs). The intercellular space of intestinal epithelial cells (IEC) is naturally protected with TJ proteins which are responsible for maintaining intestinal permeability. These proteins are continuously remodeled against external stimuli consisting of microbes and antigens [52].

The HSP27 activates cell proliferation via utilizing nuclear factor-*κ*B (NF-*κ*B) signals, which regulates cell survival, proliferation, and differentiation, and suppressing NF-*κ*B-dependent apoptotic pathways [53–56]. HSP27 endorses both ubiquitin-dependent and ubiquitin-independent degradation of unfolded proteins after cellular stresses. They exhibit their effect on several apoptotic pathways upstream and downstream of the mitochondria, such as suppressing early stages of stress cell signaling, inhibiting reactive oxygen species production via using proapoptotic proteins, and/or stimulating prosurvival proteins like kinases which in turn suppressed the release of proapoptotic signals from mitochondria [53–56]. Moreover, HSPs also possess antioxidative activities and can suppress overwhelming reactive oxygen species by stimulating antioxidant enzymes [53–56]. For instance, iHSP27 may inhibit the mitochondrial release of cytochrome-c and suppress certain kinases such as c-Jun N-terminal kinase or caspase activity. In brief, iHSP27 and iHSP70 have been reported to enhance cell survival and resistance against stresses via implying numerous pathways in different cell lines.

Heat shock proteins and major histocompatibility complex (MHC) are the molecules evolved in peptide antigen presentation [57–59]. Extracellular HSPs enable contact with direct antigen-presenting cells via stimulating different receptors, such as toll-like receptors 2 and 4; CD91 exert danger signals, thereby displaying innate immune responses [57]. Extracellular HSP27 (eHSP27) has been demonstrated to have an anti-inflammatory response depending upon the type of the immune cells-23. Further, eHSP27 activates anti-inflammatory cytokines such as interleukin (IL-10) by monocytes and suppresses differentiation into mature dendritic cells and macrophages. Gut iHSPs influence the proinflammatory NF-*κ*B pathway modulated by cytokines. The iHSPs suppress NF-*κ*B signals and are reported in gut epithelial cells [60, 61]. The mechanisms could attribute the stimulation of I*κ*B*α* and suppression of phosphorylation and degradation of I*κ*B*α* protein.

### 4.1. Weaning Stress in Pigs and Ruminants

Weaning pigs experience stressful periods that can alter intestinal and immune functions resulting in influencing animal health status. Advanced tools have been employed to minimize weaning stress; however, more collective biological understanding is required to devise strategies to combat weaning stress [62]. The factors of weaning stress include physiological, environmental, and social challenges that occur when the pigs separate from the sow, thus making them vulnerable to diseases and production losses [62]. The gastrointestinal system performs various functions such as digestion and absorption of nutrients, electrolyte balance, and secretion of digestive enzymes and acts as a barrier against detrimental molecules [62]. Abrupt changes in the diet from milk to solids make pigs prone to declined growth rates [62]. A study by Montagne et al. [63] demonstrated that reducing intake of feed during postweaning may contribute to intestinal inflammation, influencing villous height and crypt depth. Pigs experience low feed intake due to the alterations in absorption capacity of the small intestine [64]. Moreover, Rao [65] highlighted different intestinal markers linked with weaning stress, effectively reducing the physiological disturbance related to weaning stress. Weaning stress is also related to the overproduction of ROS and depletion of the antioxidant system [66]. The overwhelming status of ROS interferes with cellular function and subsequently affects TJ proteins resulting in increased gut permeability [67]. Moreover, a significant impact of weaning stress in piglets has been well documented by numerous studies [68–71].

Weaning stress is a crucial step in the calf farming system, which increases live weight gain and promotes gastrointestinal development at the weaning stage [72]. Therefore, the presence of weaning stresses [73] may influence the dairy cow production system and increase calf mortality [74]. Currently, limited literature is available on the subject of physiological and immunological responses in beef and dairy calves. Studies highlighted that weaning stress along with sudden housing reduced total leukocyte count, declined *in vitro* production of interferon-gamma, and enhanced the level of acute-phase proteins than with deferring housing for 35 days postweaning. Moreover, the transition period in cows promotes neutrophilia, suppresses interferon-*γ* production, and enhances the level of acute-phase proteins that are prevalent after the postweaning period. Hence, it is a long transition suppression in immune response indicators in calves soon after weaning. Such immune biomarkers can be utilized in the future to predict the possible occurrences of weaning stress and for overcoming respiratory infections [75].

Specific methods to improve gut health in the preweaning period are essential to reduce the calves' vulnerability against enteric infections. Gut health defines several factors which attribute to maintaining disease-free status in the GIT system [76]. Modification within the gut microbiome is an essential contributor that describes the effect on gut health [76] and is regarded as a window for improving calf gut health. Neonatal calves are most vulnerable to enteric infections, which are a pivotal cause of calf death; therefore, proper attention for improving gut health in particular calf health is required [77]. For further studies on the role of gut microbiota in weaning stress of dairy calves, a well-defined article is preferred [77].

### 4.2. Gut Microbiota and Weaning Stress

In the pig industry, the gastrointestinal tract of neonatal piglets is prone to postweaning diarrhea [78, 79] and other intestinal disorders which may directly influence intestinal absorption, intestinal barrier injury, inflammation, oxidative damage, and altered microbial response [62, 78]. Previous literature revealed that the intestinal microbiome displayed a vibrant role in sustaining intestinal function and host health, while specific bacterial communities enable the capacity to suppress infection/pathogens and enhance mucosal immunity [71]. Hence, it considers a new strategy for gut microbiota modulation to promote intestinal health.

However, the weaning stress declines the specific richness of the *Lactobacillus* group and enhances *Clostridium* spp., *Prevotella* spp., *Proteobacteriaceae*, and *E. coli*, leading to the loss of microbial diversity [80]. Moreover, weaned piglets have shown diversity and composition of gut microbiota, which is largely influenced by the quantity and sources of dietary proteins or fibres provided to postweaned pigs [81]. The interaction among nutritional constituents within intestinal cells and gut microflora is essentially vital for gastrointestinal tract function [82]. It is worth noting that the nutrition pool is pivotal for the renewal and proliferation of intestinal cells and an integral part of the microbial community [83]. The pathogenic bacteria enable to utilize proper nutrients which cannot catabolize via commensal bacteria, and it promotes the expression of virulent factors, for example, *Salmonella*, and enterohemorrhagic *E. coli* possess the capability to use ethanolamine as a source of carbon or nitrogen to obtain the benefit of nutrition in a competitive environment with other microflora [83, 84]. Enterohemorrhagic *E. coli* may consume fructose to stimulate a type III secretion system that favors the adhesion of pathogenic bacteria to host enterocytes [85]. As a consequence, weaned piglets are vulnerable to intestinal inflammation and postweaning diarrhea in response to the sudden proliferation of pathogenic bacteria and lack of microbial diversity [86]. The findings of the most recent article conclude that holly polyphenol (HP) enables attenuation of LPS-mediated intestinal injury via promoting intestinal disaccharidase activities, barrier function, and short-chain fatty acid (SCFA) production and suppresses intestinal inflammation [87]. In a study by Liedel et al. [88], certain antioxidant substances revealed a significant relationship with beneficial bacteria and adverse relation with *E. coli*. However, specific substances and bacteria indicated an opposite relationship with pigs.

## 5. Polyphenols, Heat Shock Proteins, and Gut Microbiota in Weaning Stress

Dietary approaches modify HSP expression in vivo and increase host health response via targeting specific immune responses such as Tregs. Oral induction of carvacrol in mice causes enhanced expression of HSP70 in Peyer's patches and Tregs and inhibited induced arthritis in an animal model [89]. Numerous nutritional compounds have also been documented to influence HSP expression in the GI tract *in vitro* and *in vivo* [90–92]. The anthocyanin cyanidin-3-O-*β* glucopyranoside and its aglycon form, cyanidin chloride, were documented to show antioxidant effects partially via induced expression of HSP70 in Caco2 cells [93]. The same effect was also reported using naringenin at 10–100 *μ*M [94]. In addition to that, *in vitro* studies on polyphenols have documented that flavonoid quercetin at 30–100 *μ*M and others such as flavone at 150 *μ*M, kaempferol at 100 *μ*M, and genistein at 100 *μ*M are known to be the potent inhibitors of iHSPs specifically iHSP70 [95, 96]. The grape seed extract (polyphenols) was also demonstrated to suppress iHSP70 in a bovine GEC line [97], suggesting a negative impact of several polyphenols on iHSPs. Resveratrol-triggered HSP70 expression declines the temperature rise of heat shock response and prepares cells to overcome the detrimental effects of stress levels [98]. Moreover, resveratrol brings GSH in a reduced form to suppress ROS-mediated cellular damage [99] and ameliorate H_2_O_2_-induced lipid peroxidation via decreasing MDA levels and enhancing SOD activity and mitigating the intracellular expression of ROS in Caco2 cells [100]. Resveratrol-induced HO-1 signaling is pivotal for the expression of TJ proteins through suppressing PKC activity and P38 phosphorylation [100]. Further, resveratrol activated HSP that is known to be the stimulator of anti-inflammatory regulatory T cells to protect intestinal integrity. HSP stimulation blocks the NF-*κ*B stimulation via regulating I*κ*B*α* [101].

**Figure 2:**
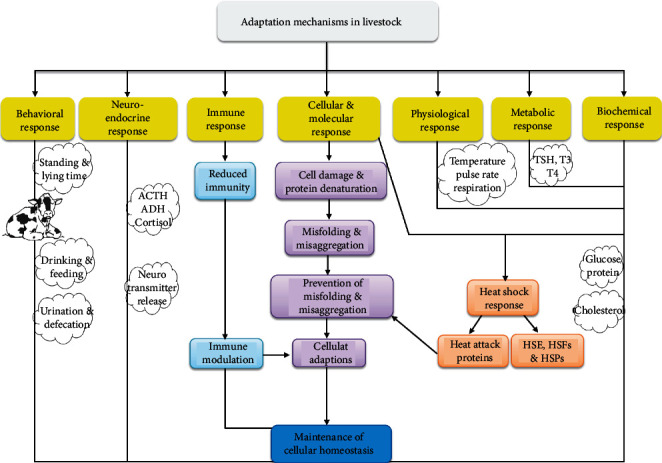
Mechanism of livestock adaptations in stress condition [149].

There are several factors to be involved in stimulating HSP expression in swine production such as high temperature, transportation, weaning, exercise, and cell exposure to toxins. The HSPs such as HSP27, HSP60, and HSP70 are overwhelmed in heat stress conditions. The expression of HSP in GIT is modified by weaning and depends upon the site of GIT and stage of postweaning [102]. Apart from that, HSPs are the conserved proteins that showed their expression in gut epithelial cells such as HSP25, HSP27, and HSP 70 and contributed to numerous cellular functions [103, 104]. It is noteworthy that iHSPs regulate barrier function via mediating TJ proteins and reverting the insult induced by oxidative and inflammatory stress on cells [26]. The intestinal and colonic epithelial cells both give similar responses against iHSP stimuli. Gut iHSP vanishes in germ-free animals [105, 106]. Further, comprehensive knowledge on the dietary intervention of heat shock proteins and gut microbiota is well documented by [51]. The animals experience different stresses during their adaptations as depicted in Figure 2.

## 6. Regulation of Polyphenols in Weaning Stress Mediated by Gut Microbiota

Nutritional research is continuously improving with particular feed additives [107]. Recently, food producers and consumers have attracted interest in promoting feed additives and prioritizing natural compounds. Polyphenols are highly effective and exhibit antimicrobial [108], antioxidative [109, 110], and antiviral [111] activities and are the large groups of natural bioactive compounds that originated from plants. The antioxidant compounds are rich in polyphenols, which can also be applied to attenuate oxidative stress in animals and enhance the antioxidant potential of animal origin products [112]. However, some scientists suggest that polyphenols do not have antioxidant capacity in the body because of their poor absorption efficacy, but they could perform other bioactivities through affecting cell signaling or microbial metabolites [113]. For example, the flavonoid could act at protein kinase and lipid kinase pathway to affect cancer and heart disease progress [114]. As for polyphenols' antioxidative activity, the polyphenol protective effect on regulation of the gut microbial community has been well deciphered by previous studies [109, 115]. The different sources of plant polyphenols have been discussed above. The vibrant activities of these compounds observed in *in vitro* and *in vivo* studies are reported to have antioxidative, immune-stimulatory, and anti-inflammatory activities [116–120].

*Eucommia ulmoides* (EU) flavone, a Chinese herbal plant, contains several compounds which provide health-enhancing effects [121]. The leaf of this plant is a rich source of flavonoids [31, 122] providing beneficial effects on health by direct scavenging of free radicals, inhibiting proinflammatory cytokines via suppressing ROS and nitric oxide, reducing inflammatory genes encompassing cyclooxygenases (COXs) and inducible nitric oxide synthase (iNOS), upregulating antioxidative enzymes, manipulating transcription factors such as NF-*κ*B and AP-1, and increasing the Nrf2 signaling pathway [123, 124]. The *in vitro* protective effect of *Eucommia ulmoides* flavones against LPS-triggered enterocyte damage (intestinal porcine epithelial cell line (IPEC-J2)) is well illustrated by Hussain et al. [120]. The inclusion of 10 *μ*g/mL EUF provided beneficial effects on cell viability, proliferation, cell cycle and apoptosis, mitochondrial bioenergetics, and NF-*κ*B protein pathway. EUF activated PI3K/AKT which serves as the cell survival and signaling pathway, ameliorated negative effects of LPS, and restored enterocyte integrity. Another study by Chun et al. [119] has shown that dietary supplementation of *Eucommia ulmoides* flavones (polyphenols) enhanced growth performance in weaned piglets which was challenged with the diquat model of oxidative stress. *Eucommia ulmoides* flavones further specified fruitful results through attenuation of oxidative stress and inflammation in intestinal morphology, reduced inflammatory cytokines, increased antioxidant response, and enhanced villi height, villus height, and crypt depth in weaned piglets.

The positive effect of tea polyphenols (TP) on a diquat-challenged model of postweaned piglets is well described by Fiesel et al. [125]. Results demonstrated that dietary TP mitigated oxidative stress and promoted growth performance to some extent. The ratio of CD4þ/CD8þ was increased suggesting the recovery of immune disruption induced by oxidative stress. Moreover, TP reduced the level of IL-1 and IFN-*γ*, which were increased by oxidative stress. However, TP enhanced the serum concentration of IL-4, indicating changes in the response of Th1 to Th2. Thus, the results showed the immunomodulatory response of TP towards weaned oxidative stress.

The coix seed associated with the family Poaceae, a rich source of nutritional compounds including polyphenols, originates in China, Japan, Thailand, and Burma [126, 127]. A study by Dairy [128] exhibited that coix seed extract significantly enhanced growth performance, promoted density and length of GIT villi, enhanced abundances of *Bacteroidetes* and genus *Lactobacillus*, and declined the richness of *Prevotella* in gut microbiota. Hence, it is a potential source of feed supplement in swine production. In a previous study, Ishihara et al. [129] used grape seed and grape marc meal extract (GSGME) or spent hops (SH) to enhance animal performance. In his experiment, pigs who were offered GSGME or SH supplement disclosed an increased gain : feed ratio, reduced levels of volatile fatty acids, and decreased counts of *Streptococcus* spp. and *Clostridium cluster XIVa* in the fecal microbiota. Further, supplementation of both groups had the lowered expression of several proinflammatory genes in the duodenum, ileum, and colon. A study by Sarker et al. [130] exhibited that plant polyphenols affected the antioxidant status of weaned piglets. Results highlighted that optimized plant polyphenol supplementation may enhance plasma antioxidants by reducing the level of MDA. Other studies by Oliveira et al. [131] revealed that plant-derived polyphenols have gut health beneficial effects. It was further indicated that polyphenols (apple and red wine pomace) had higher contents of flavonoids, implied as a feed additive, and provided beneficial effects on villi morphology and gut-associated lymphoid tissue (GALT) activation and may increase pig health. The role of polyphenols in the regulation of gut microbiota is well ascribed in Table 1.

At present, a lot of studies on weaning stress in pigs and their successful nutritional intervention strategies with polyphenol additives have been well documented, but unfortunately, such wide literature does not exist in buffalo, cattle, goats, and sheep. We tried our best to enumerate the evidence which highlights the importance of weaning stress and their possible dietary intervention with polyphenols to improve animal growth and production.

Antibiotics which are used as growth promoters can be applied to combat various postcalving health anomalies comprising preweaned heifer's death loss [132]. However, antibiotics also possess adverse effects [108]. Feed additives medicated and nonmedicated enhanced average daily gain (ADG) and declined fecal scores showing beneficial effects of natural bioactive compounds on calf health and growth. Green tea extract decreased the total number of intestinal bacteria, but the magnitude of reduction was species-specific. Beneficial bacteria Bifidobacterium spp. and Lactobacillus spp. decreased slowly, whereas C. perfringens decreased more quickly, thus improving the overall intestinal microbial balance [133]. The nonpathogenic diarrhea is caused by an imbalance in intestinal microflora, but the improved balance exerted by genotype tissue expression (GTEx) resulted in reduced diarrhea frequency. Growth performance of postweaned calf was increased by flavonoid supplementation. Calves feeding on pellet formulation consisting of fermented green tea probiotics or mixed additives reported higher ADG [134]. High tannin content and poor palatability of premium pomegranate juice (POMx) might be attributed to reduced feed intake. In this study, dry matter (DM) was not influenced; reduction in feed intake was likely contributed to decreased body weight gain (BWG) [135].

The tannin, a polyphenol derived from quebracho trees (Schinopsis lorentzii), was assumed to have protective effects on the growth and health of lambs. Results elaborated that the inclusion of tannin at 0.3% in the diet enhanced lamb growth followed by weaning. Thus, it can be employed as a feed additive during a critical period of weaning stress [136]. Growing evidence demonstrated several strategies to maintain redox homeostasis in ruminants using dietary approaches of nutraceuticals having antioxidative activities [137, 138]. Dietary polyphenols exhibit enormous health-favoring effects via regulating several mechanisms which control oxidative stress-mediated inflammation [118]. Previous literature documented that dietary supplementation of polyphenols (grape skins or juniper oil) enhanced superoxide dismutase (SOD) in cows and growing goats [139], and growing goats were provided juniper oils [140]. Further, durum wheat bran offered at 10% or 20% supplements ameliorated oxidative stress and improved antioxidative status of dairy cows and their cheese [141]. The large cell culture experiments including intestinal cells and animal models of inflammation showed the promising results of isolated polyphenol compounds or polyphenol-rich plant extracts to alleviate induced inflammation [142–145]. The anti-inflammation effect of polyphenols is regulated by cellular signaling pathways especially the most important one NF-*κ*B. Polyphenols mitigate NF-*κ*B to regulate inflammation via suppressing phosphorylation and proteasomal degradation of I*κ*B [146]. Polyphenols directly scavenge ROS and trigger the stimulation of Nrf2, thereby activating different antioxidant enzymes [147]. Direct scavenging of ROS and Nrf2 stimulation inhibits oxidative stress-mediated inflammatory pathways such as NF-*κ*B, mitogen-activated protein kinases, and activating protein 1 [148].

## 7. Conclusion

Polyphenols depict several biological activities, like antioxidant and anti-inflammation, modulating gut microbiota function and interacting with signaling pathways to revert inflammatory response. The action of polyphenols highly relies on transformation through the constituents of gut microbiota. The literature revealed the efficacy of gut microbiota transformation of specific polyphenols and determines the function of gut microbiota involved. The modulatory function of gut microbiota has been well reviewed, and its significant impact on health has also been documented. It is well rectified that polyphenols and their metabolites attribute to the maintenance of gut integrity through altering the gut microbiota balance via activation of favorable bacteria and suppression of pathogenic bacteria. Moreover, to understand the function of dietary polyphenols with gut microbiota, a combined approach of metagenomics and metabolomics is needed to dig out further insights and their positive impact on gut health.

The gut is an integral part of nutrient digestion, absorption, sensation, and regulation of intestinal immune response. When the pigs/calves are postweaned, they have a less luminal supply of nutrients making them vulnerable to intestinal tract function. Several studies have been conducted to deeply understand the significance of gut health on animal production and performance, but the description of gut health is still uniformed. The polyphenols exert multiple functions comprising antioxidant, anti-inflammation, and immunomodulatory and revert weaning effects on animals. Dietary approaches of polyphenols in weaning stress improve nutrient digestion and absorption, enhance gut barrier function, improve gut microbiota function, and thus provide beneficial effects. Most notably, host and microbial cross-talk plays an important role in maintaining intestinal homeostasis. The beneficial effects of polyphenols on intestinal function are most partially through inducing the defense and protection system by their various microbial metabolites, and HSPs are one of the most vital systems involved in the host-microbial molecular cross-talk. Meanwhile, polyphenols are a large group of plant-derived compounds, but fewer studies have been reported in weaning stress, and emphasis must be paid to more polyphenol compounds and their dietary intervention strategies against weaning stress. In addition, advanced molecular tools will be employed to figure out further insights that may help in improving the productive performance of animals.

## Figures and Tables

**Table 1:** Impact of polyphenols on gut microbiota in animal-based studies.

Compounds	Dose (animal species)	Treatment	Microbial population	Microbial population	References
Tea polyphenols	0.2% (pigs)	2 weeks	↑Lactobacilli	*Agrobacterium tumefaciens*, *P. aeruginosa*, *C. perfringens*	[146]
Low bush wild blue berries	20 g/feed/day (rats)	6 weeks	Thermomonospora spp., Corynebacteria spp., Slackia spp.	*Lactobacillus* spp., *Enterococcus* spp.	[147]
Black currant extract (leaf or berry)	30 mg/kg leaf, 13.4 mg/kg berry (3 times/week) (rats)	4 weeks	Corynebacteria spp., Lactobacilli (berry extract), Bifidobacteria (leaf and berry extract)	*—*	[148]
Resveratrol	1 mg/kg/day (rats)	25 days	Lactobacilli, Bifidobacteria	—	[149]
Apple pomace juice colloid	5% dietary suppl.	6 weeks	Bacteroidaceae	*—*	[150]
Apple juice	Free access (rats)	4 weeks	Lactobacilli, Bifidobacteria	—	[151]
Red wine polyphenols, powder	50 mg/kg (rats)	16 weeks	Lactobacilli, Bifidobacteria	Propionibacteria, Bacteroides, Clostridia	[152]
Proanthocyanidins extracted from Acacia angustissima	0.7% (low tannin diet) and 2.0% (high tannin diet) (rats)	3.5 weeks, treatment + 3.5-week washout	Bacteroides fragilis groups, Bacteroides-Prevotella-Porphyromonas groups	*C. leptum* group	[153]
Green tea	1.5 g/day (calves)	4 weeks	—	*Bifidobacterium* spp., *Lactobacillus* spp., *C. perfringens*	[133]

## Data Availability

The data supporting the review article are extracted from previous studies and have been cited.

## References

[1] Bäckhed F., Ley R. E., Sonnenburg J. L., Peterson D. A., Gordon J. I. (2005). Host-Bacterial mutualism in the human intestine. *Science*.

[2] Sommer F., Bäckhed F. (2013). The gut microbiota -- masters of host development and physiology. *Nature Reviews Microbiology*.

[3] Isaacson R., Kim H. B. (2012). The intestinal microbiome of the pig. *Animal Health Research Reviews*.

[4] Frese S. A., Parker K., Calvert C. C., Mills D. A. (2015). Diet shapes the gut microbiome of pigs during nursing and weaning. *Microbiome*.

[5] Meale S. J., Li S., Azevedo P. (2017). Weaning age influences the severity of gastrointestinal microbiome shifts in dairy calves. *Scientific Reports*.

[6] Stokes C. R. (2017). The development and role of microbial-host interactions in gut mucosal immune development. *Journal of Animal Science and Biotechnology*.

[7] Gresse R., Chaucheyras-Durand F., Fleury M. A., van de Wiele T., Forano E., Blanquet-Diot S. (2017). Gut microbiota dysbiosis in postweaning piglets: understanding the keys to health. *Trends in Microbiology*.

[8] Gentile C. L., Weir T. L. (2018). The gut microbiota at the intersection of diet and human health. *Science*.

[9] Spor A., Koren O., Ley R. (2011). Unravelling the effects of the environment and host genotype on the gut microbiome. *Nature Reviews Microbiology*.

[10] Sittipo P., Lobionda S., Lee Y. K., Maynard C. L. (2018). Intestinal microbiota and the immune system in metabolic diseases. *Journal of Microbiology*.

[11] Leamy L. J., Kelly S. A., Nietfeldt J. (2014). Host genetics and diet, but not immunoglobulin A expression, converge to shape compositional features of the gut microbiome in an advanced intercross population of mice. *Genome Biology*.

[12] Hao Y., Xing M., Gu X. (2021). Research progress on oxidative stress and its nutritional regulation strategies in pigs. *Animals*.

[13] Enríquez D., Hötzel M. J., Ungerfeld R. (2011). Minimising the stress of weaning of beef calves: a review. *Acta Veterinaria Scandinavica*.

[14] Puupponen-Pimiä R., Aura A.-M., Oksman-Caldentey K.-M. (2002). Development of functional ingredients for gut health. *Trends in Food Science & Technology*.

[15] Neveu V., Perez-Jimenez J., Vos F. Phenol-Explorer: an online comprehensive database on polyphenol contents in foods. *Database*.

[16] Andrés-Lacueva C., Medina-Remon A., Llorach R., Urpi-Sarda M., Khan N., Chiva-Blanch G. (2010). *Phenolic compounds: chemistry and occurrence in fruits and vegetables*.

[17] Appeldoorn M. M., Vincken J. P., Gruppen H., Hollman P. C. H. (2009). Procyanidin dimers A1, A2, and B2 are absorbed without conjugation or methylation from the small intestine of rats. *The Journal of Nutrition*.

[18] Bosscher D., Breynaert A., Pieters L., Hermans N. (2009). Food-based strategies to modulate the composition of the intestinal microbiota and their associated health effects. *Journal of Physiology and Pharmacology*.

[19] Manach C., Williamson G., Morand C., Scalbert A., Rémésy C. (2005). Bioavailability and bioefficacy of polyphenols in humans. I. Review of 97 bioavailability studies. *The American Journal of Clinical Nutrition*.

[20] Manach C., Scalbert A., Morand C., Rémésy C., Jiménez L. (2004). Polyphenols: food sources and bioavailability. *The American Journal of Clinical Nutrition*.

[21] Archivio M. D., Filesi C., Di Benedetto R., Gargiulo R., Giovannini C., Masella R. (2007). Polyphenols, dietary sources and bioavailability. *Annali-Istituto Superiore di Sanita*.

[22] Jacobs D. M., Gaudier E., Duynhoven J., Vaughan E. (2009). Non-digestible food ingredients, colonic microbiota and the impact on gut health and immunity: a role for metabolomics. *Current Drug Metabolism*.

[23] Hussain M. B., Hassan S., Waheed M., Javed A., Farooq M. A., Tahir A. (2019). *Bioavailability and metabolic pathway of phenolic compounds, in Plant Physiological Aspects of Phenolic Compounds*.

[24] Marín L., Miguélez E. M., Villar C. J., Lombó F. (2015). Bioavailability of dietary polyphenols and gut microbiota metabolism: antimicrobial properties. *BioMed Research International*.

[25] Lotito S., Frei B. (2006). Consumption of flavonoid-rich foods and increased plasma antioxidant capacity in humans: cause, consequence, or epiphenomenon?. *Free Radical Biology and Medicine*.

[26] Hussain T., Tan B., Murtaza G. (2020). Flavonoids and type 2 diabetes: evidence of efficacy in clinical and animal studies and delivery strategies to enhance their therapeutic efficacy. *Pharmacological Research*.

[27] Catalkaya G., Venema K., Lucini L. (2020). Interaction of dietary polyphenols and gut microbiota: microbial metabolism of polyphenols, influence on the gut microbiota, and implications on host health. *Food Frontiers*.

[28] Corrêa T. A. F., Rogero M. M., Hassimotto N. M. A., Lajolo F. M. (2019). The two-way polyphenols-microbiota interactions and their effects on obesity and related metabolic diseases. *Frontiers in Nutrition*.

[29] Gessner D. K., Ringseis R., Eder K. (2017). Potential of plant polyphenols to combat oxidative stress and inflammatory processes in farm animals. *Journal of Animal Physiology and Animal Nutrition*.

[30] The Human Microbiome Project Consortium (2012). Structure, function and diversity of the healthy human microbiome. *Nature*.

[31] Dueñas M., Cueva C., Muñoz-González I. (2015). Studies on modulation of gut microbiota by wine polyphenols: from isolated cultures to omic approaches. *Antioxidants*.

[32] Etxeberria U., Fernández-Quintela A., Milagro F. I., Aguirre L., Martínez J. A., Portillo M. P. (2013). Impact of polyphenols and polyphenol-rich dietary sources on gut microbiota composition. *Journal of Agricultural and Food Chemistry*.

[33] Moco S., Martin F. P. J., Rezzi S. (2012). Metabolomics view on gut microbiome modulation by polyphenol-rich foods. *Journal of Proteome Research*.

[34] Scalbert A., Johnson I. T., Saltmarsh M. (2005). Polyphenols: antioxidants and beyond. *The American Journal of Clinical Nutrition*.

[35] Tomás-Barberán F. A., Selma M. V., Espín J. C. (2016). Interactions of gut microbiota with dietary polyphenols and consequences to human health. *Current Opinion in Clinical Nutrition and Metabolic Care*.

[36] Mosele J. I., Macià A., Motilva M. J. (2015). Metabolic and microbial modulation of the large intestine ecosystem by non-absorbed diet phenolic compounds: a review. *Molecules*.

[37] Selma M. V., Espín J. C., Tomás-Barberán F. A. (2009). Interaction between phenolics and gut microbiota: role in human health. *Journal of Agricultural and Food Chemistry*.

[38] Mahowald M. A., Rey F. E., Seedorf H. (2009). Characterizing a model human gut microbiota composed of members of its two dominant bacterial phyla. *Proceedings of the National Academy of Sciences of the United States of America*.

[39] Ozdal T., Sela D. A., Xiao J., Boyacioglu D., Chen F., Capanoglu E. (2016). The reciprocal interactions between polyphenols and gut microbiota and effects on bioaccessibility. *Nutrients*.

[40] Most J., Penders J., Lucchesi M., Goossens G. H., Blaak E. E. (2017). Gut microbiota composition in relation to the metabolic response to 12-week combined polyphenol supplementation in overweight men and women. *European Journal of Clinical Nutrition*.

[41] Etxeberria U., Arias N., Boqué N. (2015). Reshaping faecal gut microbiota composition by the intake of *trans*-resveratrol and quercetin in high-fat sucrose diet-fed rats. *The Journal of Nutritional Biochemistry*.

[42] Li Y., Rahman S. U., Huang Y. (2020). Green tea polyphenols decrease weight gain, ameliorate alteration of gut microbiota, and mitigate intestinal inflammation in canines with high-fat- diet-induced obesity. *The Journal of Nutritional Biochemistry*.

[43] Chen L., Jiang B., Zhong C. (2018). Chemoprevention of colorectal cancer by black raspberry anthocyanins involved the modulation of gut microbiota and SFRP2 demethylation. *Carcinogenesis*.

[44] Rodríguez-Daza M. C., Daoust L., Boutkrabt L. (2020). Wild blueberry proanthocyanidins shape distinct gut microbiota profile and influence glucose homeostasis and intestinal phenotypes in high-fat high- sucrose fed mice. *Scientific Reports*.

[45] Moreno-Indias I., Sánchez-Alcoholado L., Pérez-Martínez P. (2016). Red wine polyphenols modulate fecal microbiota and reduce markers of the metabolic syndrome in obese patients. *Food & Function*.

[46] Cardona F., Andrés-Lacueva C., Tulipani S., Tinahones F. J., Queipo-Ortuño M. I. (2013). Benefits of polyphenols on gut microbiota and implications in human health. *The Journal of Nutritional Biochemistry*.

[47] Miller D. J., Fort P. E. (2018). Heat shock proteins regulatory role in neurodevelopment. *Frontiers in Neuroscience*.

[48] Beissinger M., Buchner J. (1998). How chaperones fold proteins. *Biological Chemistry*.

[49] De Maio A. (1999). Heat shock proteins: facts, thoughts, and dreams. *Shock*.

[50] Liu H., Dicksved J., Lundh T., Lindberg J. (2014). Heat shock proteins: intestinal gatekeepers that are influenced by dietary components and the gut microbiota. *Pathogens*.

[51] Schneeberger E. E., Lynch R. D. (2004). The tight junction: a multifunctional complex. *American Journal of Physiology-Cell Physiology*.

[52] Garrido C., Brunet M., Didelot C., Zermati Y., Schmitt E., Kroemer G. (2006). Heat shock proteins 27 and 70: anti-apoptotic proteins with tumorigenic properties. *Cell Cycle*.

[53] Mizushima T. (2010). HSP-dependent protection against gastrointestinal diseases. *Current Pharmaceutical Design*.

[54] Acunzo J., Andrieu C., Baylot V., So A., Rocchi P. (2014). Hsp27 as a therapeutic target in cancers. *Current Drug Targets*.

[55] Tamura Y., Torigoe T., Kukita K. (2012). Heat-shock proteins as endogenous ligands building a bridge between innate and adaptive immunity. *Immunotherapy*.

[56] Multhoff G., Pockley A. G., Streffer C., Gaipl U. S. (2012). Dual role of heat shock proteins (HSPs) in anti-tumor immunity. *Current Molecular Medicine*.

[57] Binder R. J. (2014). Functions of heat shock proteins in pathways of the innate and adaptive immune system. *The Journal of Immunology*.

[58] Carlson R. M., Vavricka S. R., Eloranta J. J. (2007). FMLP induces Hsp27 expression, attenuates NF-*κ*B activation, and confers intestinal epithelial cell protection. *American Journal of Physiology-Gastrointestinal and Liver Physiology*.

[59] Dokladny K., Lobb R., Wharton W., Ma T. Y., Moseley P. L. (2010). LPS-induced cytokine levels are repressed by elevated expression of HSP70 in rats: possible role of NF-*κ*B. *Cell Stress and Chaperones*.

[60] Campbell J. M., Crenshaw J. D., Polo J. (2013). The biological stress of early weaned piglets. *Journal of Animal Science and Biotechnology*.

[61] McCracken B. A., Spurlock M. E., Roos M. A., Zuckermann F. A., Gaskins H. R. (1999). Weaning anorexia may contribute to local inflammation in the piglet small intestine. *The Journal of Nutrition*.

[62] Pluske J. R., Hampson D. J., Williams I. H. (1997). Factors influencing the structure and function of the small intestine in the weaned pig: a review. *Livestock Production Science*.

[63] Montagne L., Boudry G., Favier C., Huërou-Luron I. L., Lallès J. P., Sève B. (2007). Main intestinal markers associated with the changes in gut architecture and function in piglets after weaning. *British Journal of Nutrition*.

[64] Zhu L., Zhao K. L., Chen X. L., Xu J. X. (2012). Impact of weaning and an antioxidant blend on intestinal barrier function and antioxidant status in pigs. *Journal of Animal Science*.

[65] Rao R. (2008). Oxidative stress-induced disruption of epithelial and endothelial tight junctions. *Frontiers in Bioscience: a Journal and Virtual Library*.

[66] Jayaraman B., Nyachoti C. M. (2017). Husbandry practices and gut health outcomes in weaned piglets: a review. *Animal Nutrition*.

[67] Moeser A. J., Pohl C. S., Rajput M. (2017). Weaning stress and gastrointestinal barrier development: implications for lifelong gut health in pigs. *Animal Nutrition*.

[68] Xiong X., Tan B., Song M. (2019). Nutritional intervention for the intestinal development and health of weaned pigs. *Frontiers in Veterinary Science*.

[69] Heo J., Opapeju F. O., Pluske J. R., Kim J. C., Hampson D. J., Nyachoti C. M. (2013). Gastrointestinal health and function in weaned pigs: a review of feeding strategies to control post-weaning diarrhoea without using in-feed antimicrobial compounds. *Journal of Animal Physiology and Animal Nutrition*.

[70] Eckert E., Brown H. E., Leslie K. E., DeVries T. J., Steele M. A. (2015). Weaning age affects growth, feed intake, gastrointestinal development, and behavior in Holstein calves fed an elevated plane of nutrition during the preweaning stage. *Journal of Dairy Science*.

[71] Kim M.-H., Yang J. Y., Upadhaya S. D., Lee H. J., Yun C. H., Ha J. K. (2011). The stress of weaning influences serum levels of acute-phase proteins, iron-binding proteins, inflammatory cytokines, cortisol, and leukocyte subsets in Holstein calves. *Journal of Veterinary Science*.

[72] Lynch E., McGee M., Earley B. (2019). Weaning management of beef calves with implications for animal health and welfare. *Journal of Applied Animal Research*.

[73] Bischoff S. C. (2011). Gut health': a new objective in medicine?. *BMC Medicine*.

[74] Malmuthuge N., Guan L. L. (2017). Understanding the gut microbiome of dairy calves: Opportunities to improve early-life gut health. *Journal of Dairy Science*.

[75] Smith F., Clark J. E., Overman B. L. (2010). Early weaning stress impairs development of mucosal barrier function in the porcine intestine. *American Journal of Physiology-Gastrointestinal and Liver Physiology*.

[76] Yin J., Wu M., Xiao H. (2014). Development of an antioxidant system after early weaning in piglets. *Journal of Animal Science*.

[77] Konstantinov S. R., Awati A. A., Williams B. A. (2006). Post-natal development of the porcine microbiota composition and activities. *Environmental Microbiology*.

[78] Rist V., Weiss E., Eklund M., Mosenthin R. (2013). Impact of dietary protein on microbiota composition and activity in the gastrointestinal tract of piglets in relation to gut health: a review. *Animal: an International Journal of Animal Bioscience*.

[79] Carmody R. N., Turnbaugh P. J. (2014). Host-microbial interactions in the metabolism of therapeutic and diet-derived xenobiotics. *The Journal of Clinical Investigation*.

[80] Zhou J., Xiong X., Wang K., Zou L., Lv D., Yin Y. (2017). Ethanolamine metabolism in the mammalian gastrointestinal tract: mechanisms, patterns, and importance. *Current Molecular Medicine*.

[81] Thiennimitr P., Winter S. E., Winter M. G. (2011). Intestinal inflammation allows Salmonella to use ethanolamine to compete with the microbiota. *Proceedings of the National Academy of Sciences*.

[82] Pacheco A. R., Curtis M. M., Ritchie J. M. (2012). Fucose sensing regulates bacterial intestinal colonization. *Nature*.

[83] Winter S. E., Winter M. G., Xavier M. N. (2013). Host-derived nitrate boosts growth of E. coli in the inflamed gut. *Science*.

[84] Xu X., Hua H., Wang L. (2020). Holly polyphenols alleviate intestinal inflammation and alter microbiota composition in lipopolysaccharide-challenged pigs. *British Journal of Nutrition*.

[85] Xu J., Xu C., Chen X. (2014). Regulation of an antioxidant blend on intestinal redox status and major microbiota in early weaned piglets. *Nutrition*.

[86] Wieten L., van der Zee R., Spiering R. (2010). A novel heat-shock protein coinducer boosts stress protein Hsp70 to activate T cell regulation of inflammation in autoimmune arthritis. *Arthritis and Rheumatism*.

[87] Ren H., Musch M. W., Kojima K., Boone D., Ma A., Chang E. B. (2001). Short-chain fatty acids induce intestinal epithelial heat shock protein 25 expression in rats and IEC 18 cells. *Gastroenterology*.

[88] Liedel J. L., Guo Y., Yu Y. (2011). Mother's Milk-Induced Hsp70 Expression Preserves Intestinal Epithelial Barrier Function in an Immature Rat Pup Model. *Pediatric Research*.

[89] Wu X., Ruan Z., Gao Y. (2010). Dietary supplementation with L-arginine or N-carbamylglutamate enhances intestinal growth and heat shock protein-70 expression in weanling pigs fed a corn- and soybean meal-based diet. *Amino Acids*.

[90] Renis M., Calandra L., Scifo C. (2008). Response of cell cycle/stress-related protein expression and DNA damage upon treatment of CaCo2 cells with anthocyanins. *British Journal of Nutrition*.

[91] Noda S., Tanabe S., Suzuki T. (2013). Naringenin enhances intestinal barrier function through the expression and cytoskeletal association of tight junction proteins in Caco-2 cells. *Molecular Nutrition & Food Research*.

[92] Hosokawa N., Hirayoshi K., Nakai A. (1990). Flavonoids inhibit the expression of heat shock proteins. *Cell Structure and Function*.

[93] Hosokawa N., Hirayoshi K., Kudo H. (1992). Inhibition of the activation of heat shock factor in vivo and in vitro by flavonoids. *Molecular and Cellular Biology*.

[94] Li X., Yang Y., Liu S., Yang J., Chen C., Sun Z. (2014). Grape seed extract supplementation attenuates the heat stress-induced responses of jejunum epithelial cells in Simmental × Qinchuan steers. *British Journal of Nutrition*.

[95] Putics A., Végh E. M., Csermely P., Sőti C. (2008). Resveratrol induces the heat-shock response and protects human cells from severe heat stress. *Antioxidants & Redox Signaling*.

[96] Burkitt M. J., Duncan J. (2000). Effects of _trans_ -Resveratrol on Copper-Dependent Hydroxyl-Radical Formation and DNA Damage: Evidence for Hydroxyl-Radical Scavenging and a Novel, Glutathione-Sparing Mechanism of Action. *Archives of Biochemistry and Biophysics*.

[97] Wang N., Han Q., Wang G. (2016). Resveratrol protects oxidative stress-induced intestinal epithelial barrier dysfunction by upregulating heme oxygenase-1 expression. *Digestive Diseases and Sciences*.

[98] Yoo C. G., Lee S., Lee C. T., Kim Y. W., Han S. K., Shim Y. S. (2000). Anti-inflammatory effect of heat shock protein induction is related to stabilization of I*κ*B*α* through preventing I*κ*B kinase activation in respiratory epithelial cells. *The Journal of Immunology*.

[99] Sarma H., Puro K., Kumar A., Mahanta N., Das M., Dewry R. (2016). Impact of heat shock protein (hsp) expression in swine: a review. *Journal of Cell and Tissue Research*.

[100] van Eden W. (2015). Diet and the anti-inflammatory effect of heat shock proteins, endocrine, metabolic & immune disorders-drug targets (formerly current drug targets-immune). *Endocrine & Metabolic Disorders*.

[101] Arnal M.-E., Lallès J. P. (2016). Gut epithelial inducible heat-shock proteins and their modulation by diet and the microbiota. *Nutrition Reviews*.

[102] Arvans D. L., Vavricka S. R., Ren H. (2005). Luminal bacterial flora determines physiological expression of intestinal epithelial cytoprotective heat shock proteins 25 and 72. *American Journal of Physiology-Gastrointestinal and Liver Physiology*.

[103] Lupp C., Robertson M. L., Wickham M. E. (2007). Host-mediated inflammation disrupts the intestinal microbiota and promotes the overgrowth of Enterobacteriaceae. *Cell Host & Microbe*.

[104] Sosnowska A., Kawęcka M., Jacyno E., Kołodziej-Skalska A., Kamyczek M., Matysiak B. (2011). Effect of dietary vitamins E and C supplementation on performance of sows and piglets, Acta Agriculturae Scandinavica. *Section A-Animal Science*.

[105] Wu S., Hu R., Nakano H. (2018). Modulation of gut microbiota by Lonicera caerulea L. berry polyphenols in a mouse model of fatty liver induced by high fat diet. *Molecules*.

[106] Lipiński K., Mazur M., Antoszkiewicz Z., Purwin C. (2017). Polyphenols in monogastric Nutrition – A review. *Annals of Animal Science*.

[107] Hu R., He Y., Arowolo M., Wu S., He J. (2019). Polyphenols as potential attenuators of heat stress in poultry production. *Antioxidants*.

[108] Dai X.-Q., Cai W. T., Wu X., Chen Y., Han F. M. (2017). Protocatechuic acid inhibits hepatitis B virus replication by activating ERK1/2 pathway and down-regulating HNF4*α* and HNF1*α in vitro*. *Life Sciences*.

[109] Wallace R., Oleszek W., Franz C. (2010). Dietary plant bioactives for poultry health and productivity. *British Poultry Science*.

[110] Vasta V., Daghio M., Cappucci A. (2019). _Invited review:_ Plant polyphenols and rumen microbiota responsible for fatty acid biohydrogenation, fiber digestion, and methane emission: Experimental evidence and methodological approaches. *Journal of Dairy Science*.

[111] Alonso Á. M., Guillén D. A., Barroso C. G., Puertas B., García A. (2002). Determination of antioxidant activity of wine byproducts and its correlation with polyphenolic content. *Journal of Agricultural and Food Chemistry*.

[112] Viveros A., Chamorro S., Pizarro M., Arija I., Centeno C., Brenes A. (2011). Effects of dietary polyphenol-rich grape products on intestinal microflora and gut morphology in broiler chicks. *Poultry Science*.

[113] Stauth D. (2007). Studies force new view on biology of flavonoids. *Free Radical Biology & Medicine*.

[114] Williams R. J., Spencer J. P., Rice-Evans C. (2004). Flavonoids: antioxidants or signalling molecules?. *Free Radiccal Biology & Medicine*.

[115] Hussain T., Tan B., Yin Y., Blachier F., Tossou M. C. B., Rahu N. (2016). Oxidative stress and inflammation: what polyphenols can do for us?. *Oxidative medicine and cellular longevity*.

[116] Yuan D., Hussain T., Tan B., Liu Y., Ji P., Yin Y. (2017). The evaluation of antioxidant and anti-inflammatory effects of Eucommia ulmoides flavones using diquat-challenged piglet models. *Oxidative Medicine and Cellular Longevity*.

[117] Hussain T., Yuan D., Tan B. (2020). *Eucommia ulmoides* flavones (*EU* F) abrogated enterocyte damage induced by LPS involved in NF-*κ*B signaling pathway. *Toxicology In Vitro*.

[118] Qu D., Sun W., Liu M., Liu Y., Zhou J., Chen Y. (2016). Bitargeted microemulsions based on coix seed ingredients for enhanced hepatic tumor delivery and synergistic therapy. *International Journal of Pharmaceutics*.

[119] Chun O. K., Chung S. J., Song W. O. (2007). Estimated dietary flavonoid intake and major food sources of U.S. adults. *The Journal of Nutrition*.

[120] Nagle D. G., Ferreira D., Zhou Y. D. (2006). Epigallocatechin-3-gallate (EGCG): chemical and biomedical perspectives. *Phytochemistry*.

[121] Ribeiro D., Freitas M., Lima J. L. F. C., Fernandes E. (2015). Proinflammatory pathways: the modulation by flavonoids. *Medicinal Research Reviews*.

[122] Deng Q., Xu J., Yu B. (2010). Effect of dietary tea polyphenols on growth performance and cell-mediated immune response of post-weaning piglets under oxidative stress. *Archives of Animal Nutrition*.

[123] Yu-Qing L. (2010). Study progress on the pharmacological functions of coix seed. *Journal of Anhui Agricultural Sciences*.

[124] Li Z., Lin Z., Lu Z. (2019). Coix seed improves growth performance and productivity in post-weaning pigs by reducing gut pH and modulating gut microbiota. *AMB Express*.

[125] Fiesel A., Gessner D. K., Most E., Eder K. (2014). Effects of dietary polyphenol-rich plant products from grape or hop on pro-inflammatory gene expression in the intestine, nutrient digestibility and faecal microbiota of weaned pigs. *BMC Veterinary Research*.

[126] Zhang H. J., Jiang X. R., Mantovani G. (2014). Modulation of plasma antioxidant activity in weaned piglets by plant polyphenols. *Italian Journal of Animal Science*.

[127] Sehm J., Lindermayer H., Dummer C., Treutter D., Pfaffl M. W. (2007). The influence of polyphenol rich apple pomace or red-wine pomace diet on the gut morphology in weaning piglets. *Journal of Animal Physiology and Animal Nutrition*.

[128] Dairy U. (2010). *Heifer Calf Health and Management Practices on US Dairy Operations*.

[129] Ishihara N., Chu D. C., Akachi S., Juneja L. R. (2001). Improvement of intestinal microflora balance and prevention of digestive and respiratory organ diseases in calves by green tea extracts. *Livestock Production Science*.

[130] Sarker M. S. K., Ko S. Y., Lee S. M., Kim G. M., Choi J. K., Yang C. J. (2010). Effect of different feed additives on growth performance and blood profiles of Korean Hanwoo calves. *Asian-Australasian Journal of Animal Sciences*.

[131] Oliveira R., Narciso C., Bisinotto R. (2010). Effects of feeding polyphenols from pomegranate extract on health, growth, nutrient digestion, and immunocompetence of calves. *Journal of Dairy Science*.

[132] Lucianer E., Schafer da Silva A., Cazarotto C. J. (2019). Addition of tannin in lamb diets after weaning: impact on performance and hematological and biochemical variables. *Acta Scientiae Veterinariae*.

[133] Oh J., Wall E., Bravo D., Hristov A. (2017). Host-mediated effects of phytonutrients in ruminants: A review. *Journal of Dairy Science*.

[134] Colitti M., Stefanon B., Gabai G., Gelain M., Bonsembiante F. (2019). Oxidative stress and nutraceuticals in the modulation of the immune function: current knowledge in animals of veterinary interest. *Antioxidants*.

[135] Stefanon B., Sgorlon S., Gabai G. (2005). Usefulness of nutraceutics in controlling oxidative stress in dairy cows around parturition. *Veterinary Research Communications*.

[136] Yesilbag D., Biricik H., Cetin I. (2017). Effects of juniper essential oil on growth performance, some rumen protozoa, rumen fermentation and antioxidant blood enzyme parameters of growing Saanen kids. *Journal of Animal Physiology and Animal Nutrition*.

[137] Bonanno A., di Grigoli A., Todaro M. (2019). Improvement of oxidative status, milk and cheese production, and food sustainability indexes by addition of durum wheat bran to dairy cows’ diet. *Animals*.

[138] Martín A. R., Villegas I., la Casa C., de la Lastra C. A. (2004). Resveratrol, a polyphenol found in grapes, suppresses oxidative damage and stimulates apoptosis during early colonic inflammation in rats. *Biochemical Pharmacology*.

[139] Biasi F., Astegiano M., Maina M., Leonarduzzi G., Poli G. (2011). Polyphenol supplementation as a complementary medicinal approach to treating inflammatory bowel disease. *Current Medicinal Chemistry*.

[140] Gessner D., Ringseis R., Siebers M. (2012). Inhibition of the pro-inflammatory NF-*κ*B pathway by a grape seed and grape marc meal extract in intestinal epithelial cells. *Journal of Animal Physiology and Animal Nutrition*.

[141] Gupta S. C., Tyagi A. K., Deshmukh-Taskar P., Hinojosa M., Prasad S., Aggarwal B. B. (2014). Downregulation of tumor necrosis factor and other proinflammatory biomarkers by polyphenols. *Archives of Biochemistry and Biophysics*.

[142] Vendrame S., Klimis-Zacas D. (2015). Anti-inflammatory effect of anthocyanins via modulation of nuclear factor-*κ*B and mitogen-activated protein kinase signaling cascades. *Nutrition Reviews*.

[143] Na H.-K., Surh Y. J. (2008). Modulation of Nrf2-mediated antioxidant and detoxifying enzyme induction by the green tea polyphenol EGCG. *Food and Chemical Toxicology*.

[144] Chuang C.-C., McIntosh M. K. (2011). Potential mechanisms by which polyphenol-rich grapes prevent obesity-mediated inflammation and metabolic diseases. *Annual Review of Nutrition*.

[145] Archana P., Aleena J., Pragna P., Vidya M., Niyas A., Bagath M. (2017). Role of heat shock proteins in livestock adaptation to heat stress. *J Dairy Veterinary Animal Research*.

[146] Hara H., Orita N., Hatano S. (1995). Effect of tea polyphenols on fecal flora metabolic products of pigs. *Journal of Veterinary Medical Science*.

[147] Lacombe A., Li R. W., Klimis-Zacas D. (2013). Lowbush wild blueberries have the potential to modify gut microbiota and xenobiotic metabolism in the rat colon. *PLoS One*.

[148] Molan A.-L., Liu Z., Kruger M. (2010). The ability of blackcurrant extracts to positively modulate key markers of gastrointestinal function in rats. *World Journal of Microbiology and Biotechnology*.

[149] Larrosa M., Yañéz-Gascón M. J., Selma M. V. (2009). Effect of a Low Dose of Dietary Resveratrol on Colon Microbiota, Inflammation and Tissue Damage in a DSS-Induced Colitis Rat Model. *Journal of Agricultural and Food Chemistry*.

[150] Sembries S., Dongowski G., Jacobasch G., Mehrländer K., Will F., Dietrich H. (2003). Effects of dietary fibre-rich juice colloids from apple pomace extraction juices on intestinal fermentation products and microbiota in rats. *British Journal of Nutrition*.

[151] Sembries S., Dongowski G., Mehrländer K., Will F., Dietrich H. (2006). Physiological effects of extraction juices from apple, grape, and red beet pomaces in rats. *Journal of Agricultural and Food Chemistry*.

[152] Dolara P., Luceri C., De Filippo C. (2005). Red wine polyphenols influence carcinogenesis, intestinal microflora, oxidative damage and gene expression profiles of colonic mucosa in F344 rats. *Mutation Research-Fundamental and Molecular Mechanisms of Mutagenesis*.

[153] Smith A. H., Mackie R. I. (2004). Effect of condensed tannins on bacterial diversity and Metabolic activity in the rat gastrointestinal tract. *Applied and Environmental Microbiology*.

